# 3D Holographic Observatory for Long-term Monitoring of Complex Behaviors in Drosophila

**DOI:** 10.1038/srep33001

**Published:** 2016-09-08

**Authors:** S. Santosh Kumar, Yaning Sun, Sige Zou, Jiarong Hong

**Affiliations:** 1 Department of Mechanical Engineering, University of Minnesota, Minneapolis, MN 55455, USA; 2 Saint Anthony Falls Laboratory, University of Minnesota, Minneapolis, MN 55414, USA; 3 Translational Gerontology Branch, National Institute on Aging, Baltimore, MD 21224, USA

## Abstract

*Drosophila* is an excellent model organism towards understanding the cognitive function, aging and neurodegeneration in humans. The effects of aging and other long-term dynamics on the behavior serve as important biomarkers in identifying such changes to the brain. In this regard, we are presenting a new imaging technique for lifetime monitoring of *Drosophila* in 3D at spatial and temporal resolutions capable of resolving the motion of limbs and wings using holographic principles. The developed system is capable of monitoring and extracting various behavioral parameters, such as ethograms and spatial distributions, from a group of flies simultaneously. This technique can image complicated leg and wing motions of flies at a resolution, which allows capturing specific landing responses from the same data set. Overall, this system provides a unique opportunity for high throughput screenings of behavioral changes in 3D over a long term in Drosophila.

Understanding behavior in invertebrate model organisms like *Drosophila* is an important approach towards unraveling the effects of aging, age-related diseases, and neurodegeneration in humans^1^,^2^. These organisms serve as excellent candidates for targeted drug testing and high-throughput drug screenings due to their short lifespans, as well as similarities in genetic and behavioral responses with higher organisms^3^,^4^,^5^. However, the ubiquity of genetic and bio-molecular tools is in stark contrast to those available to capture and investigate such fine complex motions and behaviors at high resolutions at the same pace^6^. Due to such limitations, most studies on behaviors of *Drosophila*, especially over its lifespan, captured limited information such as trajectories in 2D arenas using a single camera or an infrared beam detector^7^,^8^,^9^. A number of studies have also imaged flies in 3D arenas to study wing aerodynamics and flight coordination, however over much shorter durations and using multiple cameras^10^,^11^,^12^,^13^. There are multitudes of challenges in extending such techniques towards longer time scales, ranging from the volume of data collection to the complexity of the setup involving multiple cameras.

Digital Inline Holography (DIH), primarily a microscopic imaging technique, has emerged as an effective and compact tool to obtain 3D information of objects with both high spatial resolution and extended depth of field, i.e. more than 3 orders higher than traditional microscopy, using a single camera^14^ (Supplementary Methods). A typical DIH setup (Fig. 1a) consists of a single-beam collimated light source illuminating the objects of interest and recording the interference patterns (i.e. hologram) of the scattered and unscattered portions of the beam. We can extract the information stored in a hologram through convolution with a diffraction kernel, numerically, into a 3D optical field. Furthermore, by applying appropriate segmentation algorithms we can segment the objects of interest. Combined with object tracking and high-speed imaging, DIH has been successfully applied in the study of 3D dynamics of microorganisms (e.g. zooplankton, flagellates, bacteria, and algae) in their natural environment^15^,^16^,^17^,^18^. In this paper, we propose a large field of view (FOV) imaging system based on DIH, which can be applied for long-term monitoring of fine behaviors in free-flying *Drosophila*.

Extending the microscopic technique to study the behaviors of *Drosophila* over a large FOV is challenging due to a number of technical limitations. First is the poor longitudinal resolution associated with DIH due to an extended depth of focus, which scales with the size of the object as well as the NA (numerical aperture) of the imaging system^19^. Second, the computational load involved in extracting all relevant 3D information from individual holograms is enormous, preventing its implementation for large datasets generated from long-term recordings. We have addressed both issues through specific numerical processing techniques, and presented here the experimental setup and results from recordings of a group of *Drosophila*. Finally, we will also present results to show the uncertainty of the numerical algorithm, using both simulations and experimental calibration, the details of which can be found in the Supplementary Methods.

## Hardware

**Figure 1:**
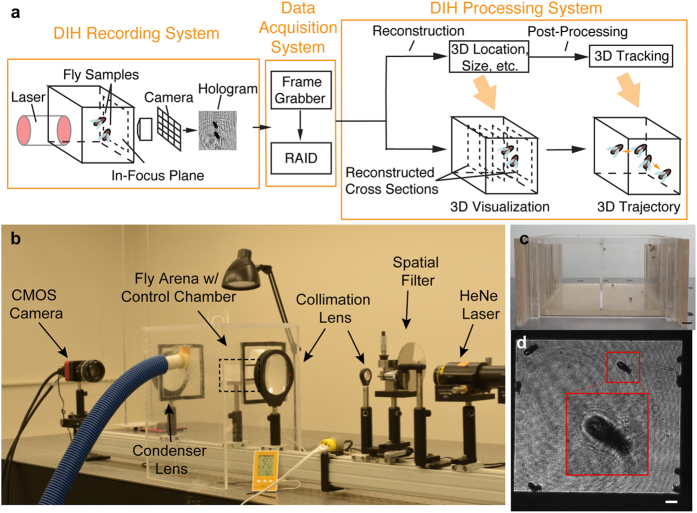
(**a**) A schematic of DIH imaging system with specific application to *Drosophila*. (**b**) DIH fly observatory setup with He-Ne laser, spatial filter, collimation lens, fly arena (marked by box) with environmental control chamber (with pipe connected to humidifier) and the CMOS camera. (**c**) Fly arena loaded with *Drosophila* and fly food in food trays on the right wall with 10 mm scale bar. Current system would allow simultaneous recording of control and experimental flies. (**d**) Camera view of the arena, an inset of a magnified holographic image of a fly marked on the front wall and a 2 mm scale bar.

Our DIH fly observatory (Fig. 1b) consists of a Helium-Neon Laser (REO Inc. R-30992 He-Ne; 12 mW; 633 nm), a spatial filter (Newport Inc. model 900; 20x objective; 15 μm pinhole) and a collimating lens (Newport Inc. Plano-convex lens; *d* = 75 mm; *f* = 300 mm), producing a clean 70 mm diameter Gaussian beam. The fly arena made of acrylic (Fig. 1c) with dimensions of 70 × 35 × 55 mm^3^ is divided into 2 separate chambers that can host up to 20 flies in each and offers minimal interference over long-term behavior studies. The arena is housed inside a control chamber also made of optically clear acrylic to maintain a stable humidity level through a humidifier connected via a flexible pipe (Fig. 1b). Along with the air conditioning system in the lab, we are able to ensure a stable temperature and humidity for the duration of the experiment. The arena is equipped with special feeding trays on opposite walls to provide nutrition to the flies over the course of the experiment, without loss of optical access (Supplementary Methods). The holograms are captured on the CMOS camera (IO Industries Flare 2M360 2k × 1k pixel^2^; 5.5 μm/pixel) through a combination of a condenser lens (Newport Inc. Plano-convex lens; *d* = 75 mm; *f* = 300 mm) and a 50 mm f/1.2 Nikon imaging lens at a resolution of 30 μm/pixel (Fig. 1d). A dedicated 56 TB RAID bay (14 × 4 TB) was designed to securely store the data captured by the frame grabber at rates up to 100 fps over long term. The proposed technique can monitor a group of flies over large time scales (~1 week with sampled recording of data at 8 hours/day) and provide a way to obtain the 3D motion information through appropriate holographic processing algorithms i.e. reconstruction and segmentation. To demonstrate the technique, we have selected a specific dataset of 2 hours with 9 flies, recorded during the hours of dusk (i.e. 6–8 pm). The numerical algorithm extracted the 3D trajectories and various motion statistics (spatial distributions, ethograms etc.) over the entire duration along with some complex wing-leg-torso coordination events that occur over shorter time scales for individual flies.

### Numerical Algorithm

The strength of the proposed technique is its ability to extract complete 3D information from data captured by a single camera with minimal hardware and simple computations. All other contemporary 3D imaging techniques involve the use of multiple cameras obtaining the position of the object through complicated stereo reconstructions, which prohibit their use over large time scales^10^,^11^,^12^,^13^. The main steps of our processing algorithm are image enhancement, 2D segmentation, 2D tracking, extraction of 3D position through numerical reconstruction using a Fresnel diffraction kernel and the post processing of results to obtain relevant behavioral measures.

**Figure 2:**
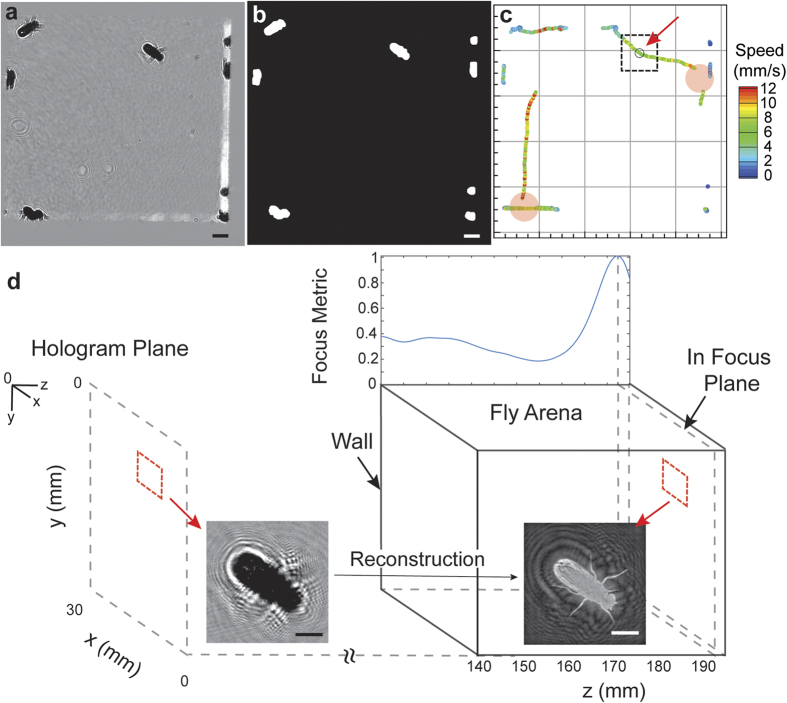
Algorithm flow chart illustrating the entire processing steps in order, using the hologram in Fig. 1d. (**a**) Hologram after enhancement (*z* = 0) through time average background subtraction. (**b**) Binary image after automatic thresholding and opening used to identify *Drosophila* in the FOV. Both images contain a scale bar of 2 mm. (**c**) 2D trajectories of detected single objects colored by speed (2D) over 2.5 s on either side of the hologram with an arrow pointing to the current time step (marked with a black circle and a ROI of size 150 × 150 pixel^2^). The red disks indicate locations and periods of missing data due to occlusion, which can be seen in Supplementary Video 3. (**d**) Illustration of the focus metric algorithm showing the recording plane with an inset of the original hologram and its reconstruction (inverted grayscale). The curve is overlaid on top of the fly arena, with a common x-axis for the z position (origin at the top left of hologram plane) and the peak corresponds to the plane of maximum focus shown by the inset of the refocused image. The arena is located at 140 mm to satisfy the paraxial approximation (Supplementary Methods). Scale bar of 2 mm on both insets.

Figure 2 illustrates our processing algorithm using a sample hologram selected from the recorded dataset (Fig. 1d). A time averaged background subtraction eliminates any noise present in the image caused due to aberrations on the imaging windows and improves the signal-to-noise ratio of the diffraction fringes (Fig. 2a). After enhancement a moving average filter is applied to smooth out variations in intensity inside the body of the fly, due to the presence of transparent wings that causes over segmentation on thresholding. An automatic threshold based on the mean value between the first two peaks of the histogram is used to segment the image in 2D as any intensity lower than this threshold belongs to a fly or random noise in the background (Supplementary Methods). A binary opening operation, with a structuring element matching the size of appendages (10-pixel disk), is performed to remove all of the limbs and wings improving the accuracy of the centroid calculation (Fig. 2b). A detection uncertainty in *xy* is about 3–5% of body length, which was estimated through sampling stationary objects at various locations on the image (Supplementary Methods). The 2D positions and the area of the object are then extracted through a binary labeling process.

The objects detected in 2D are tracked in time (Fig. 2c) in order to obtain information on the planar motions of the flies. There are several tracking algorithms available, all of which are based on the optimization of a multi-object assignment problem^20^. We employed the method of tracking by the minimization of position change (Nearest Neighbor Algorithm), where an optimal candidate assignment is made based on a greedy search technique (i.e. Hungarian Algorithm)^21^. Single view imaging results in several scenarios where multiple objects are overlapped especially in the longitudinal direction. When the duration of overlap is within a fraction of a second and involving only two objects, we can identify both when they separate, based on their velocities. However, this approach fails when the duration becomes longer or if there are more than two objects overlapping. Our algorithm automatically identifies these situations and excludes them from the calculation of statistics for single flies. This limitation can be overcome by using a two-view holographic observatory, where we capture holograms along two orthogonal directions, completely eliminating any issues with overlaps in the arena.

The next step in the processing involves depth reconstruction of the selected 2D trajectories through convolution with the diffraction kernel and segmentation to obtain the 3D locations of the objects. The image is reconstructed by convolving with the wave diffraction kernel implemented efficiently using the Fourier transform property of convolutions. Various metrics such as gradients, Laplacian, variances in intensity^22^, enclosed intensity energy^23^, or intensity profiles^24^ of the reconstructed image stack have been successfully applied to identify the location of sharpest focus. However, they all require the storage of the complete stack for each detected object, which quickly grows to overwhelm the memory available. An alternative method to the above exists, based on the maximization of an l_1_ norm of the images in the Fourier spectrum domain, which can be effectively coupled with the reconstruction step^25^ (Supplementary Methods). This method reduces the problem from a memory intensive search in the intensity images to the maximization of a 1D function in the Fourier domain. Additionally, we have modified the diffraction kernel reported to utilize the paraxial approximation (Fresnel Diffraction Kernel) to improve the computation speed. We validated the accuracy of the algorithm with synthetically created holograms as well as needle translated along the longitudinal direction with a linear stage (Supplementary Methods).

The first step in calculation of the l_1_ norm involves the computation of the Fourier transform. In order to speed up the processing, the holograms are cropped to include a region just around the object of interest of size 150 × 150 pixel^2^ and intensity rescaled to have a zero mean, before taking the Fourier transform. Furthermore, a Tukey window of size 150 × 150 is used on the cropped image and zero padded to 256 × 256 to eliminate the artifacts that arise from the sharp transition at the borders. Before the computation of the l_1_ norm, a Gaussian low pass filter is applied to suppress the high frequency information, which is mainly introduced by noise present in the image. The l_1_ norm of the product of the hologram’s spectrum and the diffraction kernel is obtained at each *z* position and the value saved. In order to achieve a resolution higher than the sampling rate in *z*, we interpolate the focus metric function with a spline. A sample l_1_ norm plot along with the input hologram and the refocused image at the location of the peak has been illustrated below (Fig. 2d). After the creation of the focus metric function, the location of the object can be identified as the position of the peak in the curve. However, there are several instances where we encounter focus metric curves with multiple prominent peaks due to the presence of other flies in close proximity, whose diffraction patters were present in the input hologram of the fly, along with noise in the signal. In order to accurately determine the peaks, a multi pass peak search algorithm has been implemented. During the first pass, we select only those peaks with a minimum specified quality and leave the remaining positions as gaps in the data which will be filled in during the post processing step using data from all time steps. The multi pass method was validated using a sequence with a fly moving in the longitudinal direction (Supplementary Methods).

The final step is post-processing to extract useful information from results reconstructed by the processing algorithm. The missing z positions are linearly interpolated from all the high quality seed values. The 3D position at each time step is then corrected using a sparse sampled velocity, calculated at intervals which correspond to displacements greater than the uncertainty in detection (Supplementary Methods). We also performed a complete uncertainty analysis for the focus metric algorithm for both stationary and moving test samples, which can be found in the Supplementary Methods as well.

### Motion Statistics based on 3D Trajectories

Some of the key insights gained include spatial distributions, ethograms, 3D trajectories and statistics on proximity to other flies over time. As stated earlier, our algorithm automatically screens for trajectories that remain overlapped in the longitudinal direction over prolonged durations and excludes them from calculation of statistics for individual flies. This is done by identifying single flies from their 2D trajectories through changes in area and proximity to neighbors over time, which are clear indicators for impending occlusion of objects. An identical process can be used in situations when objects emerge from a clustered group. The automatic algorithm identifies 50% of the total number of tracks as belonging to single objects corresponding to 5700 single trajectories of varied lengths over the entire duration (Supplementary Methods).

**Figure 3:**
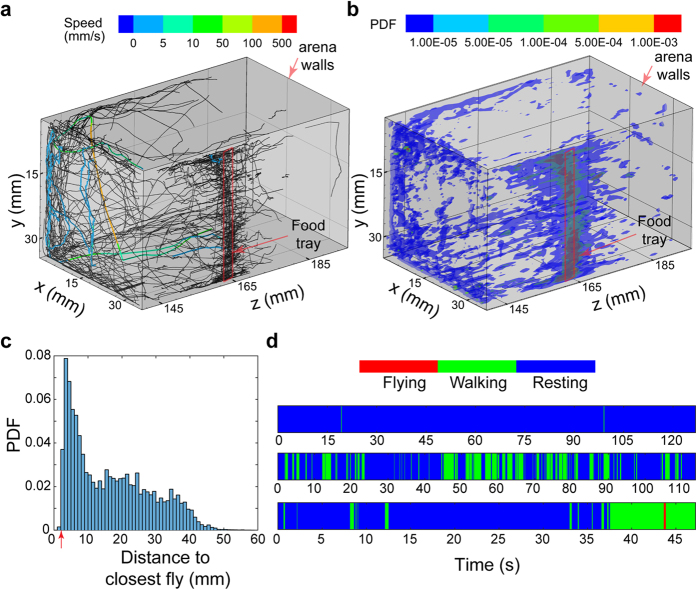
(**a**) 3D trajectories of single flies over the entire processed duration showing the captured motion patterns inside the 3D arena with walls marked in gray. Three specific trajectories have been colored with the speed illustrating a predominantly stationary, slow moving and fast moving fly. (**b**) Probability distribution of objects in 3D space over the entire duration. Location of the food trays on both chambers are marked with a red box. (**c**) Probability Density Function (PDF) of distance to the closest fly over the entire processing duration with an arrow indicating the body length of the flies. (**d**) Ethograms of motion for the three selected tracks in a showing 3 levels of motion selected based on a speed threshold.

Figure 3 showcases some of the possible long-term measures that can be obtained from the holographic data set which include 3D trajectories of single flies, a 3D spatial distribution of flies, distribution of distances to the closest fly and ethograms. The trajectories are plotted over the entire duration of processing showing the complicated 3D motions of the flies inside the chamber. We have presented trajectories which are selected based on a minimum length for clarity as a large fraction of the data set consists of short tracks. Three specific tracks have been highlighted colored by the speed of motion showcasing their contrasting motion patterns, one which involves flying motion near the top left corner, another one walking on the front wall and a third one predominantly stationary near the food tray (indicated by the red rectangle) (Fig. 3a). The spatial distribution map (Fig. 3b) is the rendering of probabilities for finding a fly at a specific location calculated using sampling bins of size 0.3 × 0.3 × 1 mm^3^. The higher probabilities along the middle of the right wall correspond to the location of the food tray, which indicates a feeding behavior for a major fraction of the processed duration. A second location with a relatively high probability is the top corner of the front wall, corresponding to a busy crossing point for flies moving away from the food wall. This along with the closest distance map, which is a distribution of the Euclidean distances to the closest fly in every frame (Fig. 3c), provides information on the clustering behavior of the group of flies at various locations of the arena. The dominant peak in the distribution is located close to the body length of the flies, which would occur when multiple flies stay close to the food for long durations. The 3D distribution and the closest distance map together provide an effective insight into the group dynamics of the flies. The ethograms of motion for the three selected tracks from Fig. 3a were created with three levels of motion (Supplementary Methods) namely, resting, walking and flying (Fig. 3d). The ethograms are in the order of increasing speeds of motion and are progressively shorter in length. The first of the three, about 2 minutes long can be found on the sidewall near the food. The second, also about the same length as the first, is moving on the front wall close to the camera. The fastest trajectory includes a stationary phase at the beginning followed by motion along the wall, a vertical fall and a continuation of the walking phase along the wall. Ethograms provide us with characteristic motion patterns of individual flies which can serve as an important physical biomarker in various genetic and biochemical studies^1^,^2^. The general distribution shows that the flies are stationary over long durations of the processing time. The variation in climbing strength with aging, which can be inferred from the 3D trajectories, over long time scales have also been shown to be an effective marker for neurodegeneration^23^.

### Complex Behaviors

**Figure 4:**
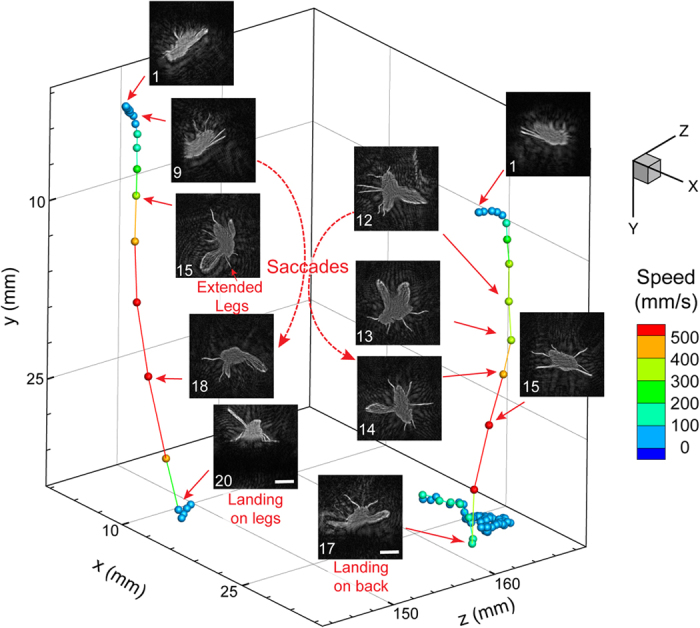
In-focus reconstructions of sequences illustrating two separate 3D trajectories showcasing different landing responses. (i) Landing response with a single saccade and landing on its feet. (ii) Collision avoidance response undergoing multiple quick saccades and falling on its back. All numbers are frame positions relative to the first frame, recorded at 100 fps with a scale bar of 2 mm and colored by speed. All images have been inverted in order to differentiate from the white background.

Apart from the trajectory-based motion statistics, the developed imaging system is also capable of identifying complex motions like flying, falling, climbing etc. at recording resolutions. It is worth noting that these complex behaviors do not have an appreciable signature in the trajectory-based motion statistics and are not captured in other aging-related long-term study of fly behaviors^26^. As a test case, we have successfully identified quick vertical motions through an appropriate velocity threshold (Supplementary Methods). The reconstruction algorithm processes these specific time sequences and creates videos that capture complex motions of legs, wings and torso from the same holographic data set. Two specific examples of such motions have been plotted along with snapshots to illustrate specific landing responses associated with flies, where the resolution of our technique enables us to capture very complicated leg motions from the single view of the hologram. The motion of a fly towards a surface consists of straight line trajectories coupled with quick turns (saccades) and for a typical landing event, the rate of expansion of the image seen by the fly (visual stimulus) causes either a collision avoidance or landing (Fig. 4) responses^27^. These are primarily characterized by the position of the forelegs at time steps before the moment of impact. The fly undergoing the collision avoidance response manages multiple saccades before hitting the bottom surface, whereas the other one has a single saccade and manages to land on its feet (Supplementary Video 1 & 2). The reconstructed images enable us to extract with good accuracy the orientation of the legs and wings with respect to the body as well as center of rotation for the saccades. These particular image and video sequences showcase the power of the holographic technique in obtaining images of complex 3D motions over large depth of fields through low cost numerical schemes.

## Discussion

Here we introduce a new imaging system and processing algorithm for long-term study of complex behaviors of a group of freely moving *Drosophila* based on digital inline holography. We have showcased some motion statistics like ethograms, spatial distributions and 3D trajectories that can be obtained along with complex behaviors when flies undergo specific landing responses. The novelty of our technique lies in its ability to combine a simple and compact inline holographic imaging system with robust image processing algorithms to chart both the observable complex fly behaviors and unobservable statistical behavioral changes over durations of a few weeks. With further improvements on computational speed and autonomous behavior identification based on machine learning algorithms, we could obtain a real-time holographic fly observatory which will eliminate the need for large data transfer and storage. This would give us an opportunity to observe changes in the behavior over the lifespan of flies, with minimal data collection, which can be vital for fields like gerontology and high throughput drug testing. Differential screenings of separate sets of flies can be carried out simultaneously through the current design of the fly arena with separate chambers as shown in our setup. Such efforts would open up a vast array of phenotype and genotype identifications towards the study of social behaviors from simple observation-based experiments. The goal of such an endeavor would be a fully automated system for behavior studies in *Drosophila*, as well as any other small invertebrate model organisms of interest e.g. roundworm, nematode, zebrafish etc., over durations of their lifespan in 3D environments.

## Methods

### Fly maintenance and experimental animal preparation

Fruit flies (wild-type strain *Canton S*), obtained from our collaborators at NIH, were maintained on standard cornmeal agar medium at 23 °C, 55% humidity and a 12 h light/dark cycle in an incubator. To get experimental flies, adult flies of mixed sex were collected within 24 h after eclosion and mated for 24 h. Female flies were then sorted out and placed into vials containing the medium with sugar and yeast extract at the 1:1 ratio (SY1:1) with up to 40 flies per vial^28^. Experimental flies were aged in the vials for 3 days, and later transferred to the recording arena to perform the experiment.

### Environmental Control

An environmental box made out of clear acrylic was constructed to maintain a stable humidity. Using a humidifier attached to the chamber, a relative humidity of 50–60% was maintained for ideal fly housing conditions and to prevent drying of food. Temperature of the lab was controlled by a central air conditioning system, which maintained the lab close to 23 °C over the entire course of the experiment. Nutrition for the experimental flies is provided through specially designed food tray located on the far walls of arena. The SY 1:1 food is replaced every 24 hours in order to ensure proper food quality and moisture levels.

### Software

All of the codes used to process the data was written using MATLAB. We have included a pseudo code used for the processing of our data in the Supplementary Methods.

## Additional Information

**How to cite this article**: Kumar, S. S. *et al*. 3D Holographic Observatory for Long-term Monitoring of Complex Behaviors in Drosophila. *Sci. Rep.* **6**, 33001; doi: 10.1038/srep33001 (2016).

## Supplementary Material

Supplementary Video 1

Supplementary Video 2

Supplementary Video 3

Supplementary Video 4

Supplementary Information
