# Supplementary material for: 3D Holographic Observatory for Long-term Monitoring of Complex Behaviors in Drosophila
Source: Sci Rep. 2016 Sep 8;6:33001. doi: 10.1038/srep33001 (PMC5015086; doi:10.1038/srep33001)
Supplement: Supplementary Information [file srep33001-s5.pdf]

3D Holographic Observatory for Long-term Monitoring of Complex Behaviors in Drosophila

S. Santosh Kumar [1,2] Yaning Sun[3], Sige Zou[3], Jiarong Hong[1,2] *

1. Department of Mechanical Engineering, University of Minnesota, Minneapolis, MN 55455, USA

2. Saint Anthony Falls Laboratory, University of Minnesota, Minneapolis, MN 55414, USA

3. Translational Gerontology Branch, National Institute on Aging, Baltimore, MD 21224, USA

* corresponding author (jhong@umn.edu)

Digital Inline Holography

Digital Inline Holography (DIH) is an imaging technique capable of capturing the 3D information of the objects using a single camera (for an in-depth review of DIH refer to [1]). DIH obtains 3D information by recording both the amplitude and phase of the light through interference. As shown in **Supplementary Fig. 1a**, the light incident on the object scatters, forming the object wave, and interferes with the remaining unscattered portion, called the reference wave. The interference produces modulation of intensity, shown as concentric rings around the object that encode the size (low frequency) and depth information (high frequency). The information in these patterns (fringes), can be decoded through numerical reconstruction to obtain sharply focused images of objects that are out of focus in the recorded holograms.

A typical DIH system (**Supplementary Fig. 1b**) consists of a coherent light source to generate the interference between the object and reference beams. The light passes through a spatial filter, with an objective lens to focus the beam through a pinhole, performing an optical low pass filter. After the spatial filter, a convex lens collimates the beam to create a planar wave front which is then incident on the object of interest. The use of planar waves allows us to extend the length of the test section without loss of laser intensity, at any size of beam diameter used.

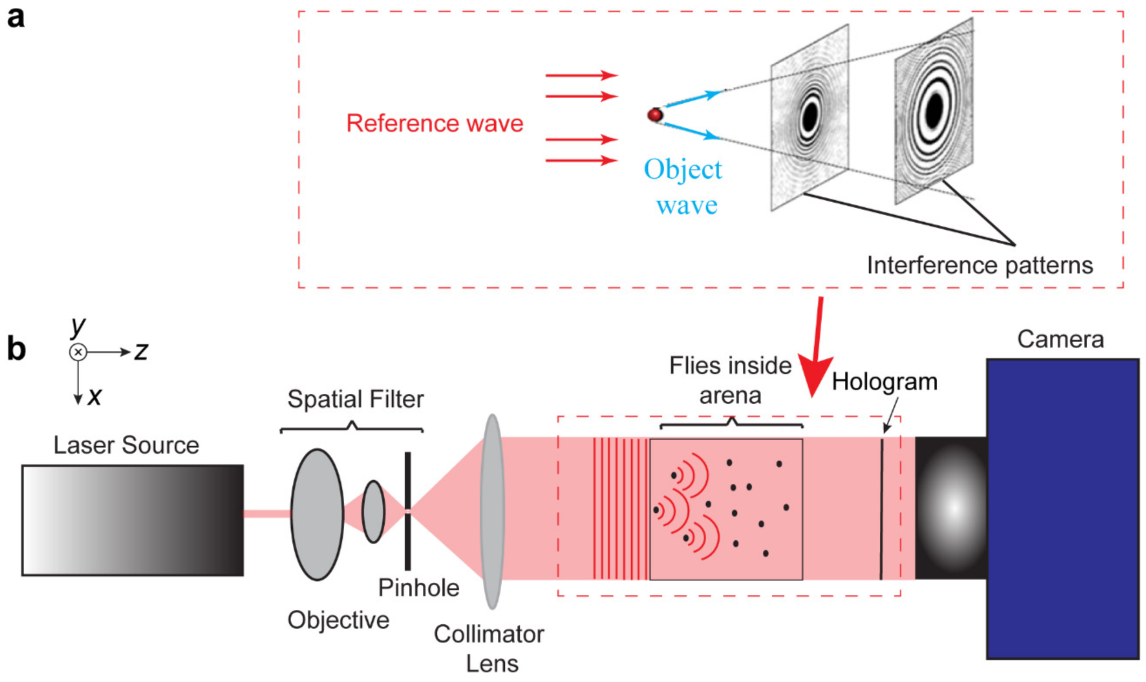

Supplementary Figure 1: (**a**) Illustration of the hologram formation from the interference between object wave and reference wave in a DIH setup. (**b**) A schematic of DIH imaging system with a collimated laser source.

Once the hologram is recorded, it is numerically reconstructed into a 3D image consisting of a stack of 2D image slices. This reconstruction process is achieved through convolution with optical diffraction kernel, e.g. commonly-used Fresnel diffraction

kernel (**Supplementary Equation 1**), which represents the point spread function for holographic imaging.

$$I(x, y, z = z_{\text{recon}}) = |I_0(x, y) * h(x, y, z)|, \qquad h(x, y, z) = \exp\left\{\frac{ik}{2z}((x - x_o)^2 + (y - y_o)^2)\right\}$$

Supplementary Equation 1: Numerical reconstruction of a hologram at $z = z_{\text{recon}}$ through the convolution of the recorded hologram (I_0) with the Fresnel diffraction kernel ($h\ (x, y, z)$). (x_0, y_0) is the center of the hologram plane.

To increase the computational speed, the reconstruction is often performed using the Fourier ($\mathcal{F}\{\cdot\}$) and inverse Fourier ($\mathcal{F}^{-1}\{\cdot\}$) transformation shown below.

$$I(x, y, z) = |\mathcal{F}^{-1}\{\mathcal{F}\{I_0(x, y)\} \times H(f_x, f_y, z)\}|, \quad H(f_x, f_y, z) = \exp\{-i\pi\lambda z(f_x^2 + f_y^2)\}$$

Supplementary Equation 2: Numerical reconstruction based on Fourier transformation. $H(f_x, f_y)$ is the Fourier transform of Fresnel diffraction kernel.

The **Supplementary Figure 2** illustrates the numerical reconstruction process with a schematic and a sample reconstructions of a fruit fly hologram. The original hologram recorded at z=0 (**Supplementary Figure 2b**) can be numerically reconstructed to a series of 2D images corresponding to different z locations. Once the z value in $H(f_x, f_y, z)$ coincides with the actual location of the object, an in-focus image of the object is obtained (e.g. **Supplementary Figure 2e**).

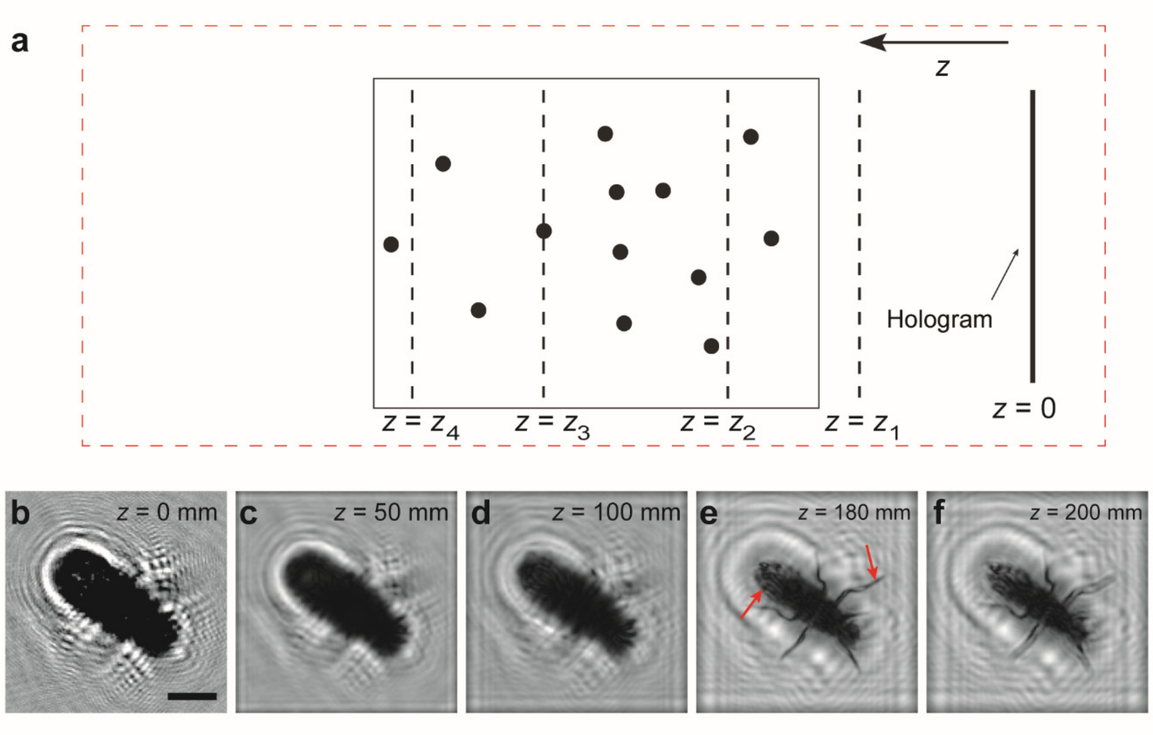

Supplementary Figure 2: (**a**) A schematic showing the numerical reconstruction of 2D images at different z locations (e.g. $z = z_1$, z_2, z_3, z_4) from the recorded hologram (i.e. at z = 0). (**b**) A sample of recorded hologram and (**c-f**) the corresponding reconstructed 2D images at different z locations. Note that the object is in focus at z = 180 mm, which is identified by the sharp features of the legs and wings (marked by arrows).

Note that due to the characteristics of diffraction kernel (e.g., $H(f_x, f_y)$ being symmetric), objects located at the same distance $|z|$ from the recorded hologram plane will be reconstructed in the same manner. The feature induces ambiguity in determining the actual location of the object (i.e. z>0 or z<0). This ambiguity is usually rectified by ensuring that all the objects being studied are located on the same side of the hologram during recording.

Similar to DIH, the 3D imaging technique based on Time-of-Flight (TOF) range cameras (e.g. Hansard et al. 2012) can also achieve depth measurements at sub centimeter resolutions from a single camera[2]. The approach uses a laser (typically infrared) to illuminate the object of interest and captures the reflected beam along with the time difference between the two. The difference in time provides us the time-of-flight between the beams, which is used to calculate the distance of the object from the camera. However, compared with DIH, TOF techniques suffer from limited depth of field and poor temporal resolution. As TOF utilizes backward scattered light, weaker in intensity than forward scattered light, there is a constrain on the depth of the imaging volume. Furthermore, the weak backward scattered light requires an integration of the signal over a finite time duration, for a reliable measurement, lowering the temporal resolution of the system.

Focus Metrics using l_1 norm

The identification of the in-focus image from the image stack often involves the use of intensity based metrics such as Laplacian, gradient, variation of intensities etc. The process involves two Fourier transforms in the reconstruction process and further steps on the intensity of the slices. Given a large dataset, the computational time for this approach would quickly become exorbitant, with multiple objects per image. To overcome this limitation, we employ a frequency-based focusing method, referred to as l_1 norm Focus Metrics, introduced by Li et al. (2009)[3]. Specifically, the algorithm identifies the z value corresponding to the maximum of the l_1 norm, the absolute value of the hologram's propagation frequency spectrum, i.e. $\mathcal{F}\{I_0(x,y)\} \times H(f_x, f_y, z)$, as the in-focus location of the object. The physical basis behind this approach can be understood as follows.

Essentially, $H(f_x, f_y, z)$ represents a 2D chirp function which is used to modulate the frequencies captured in the hologram. The peak locations of this chirp function varies based on the value of z used in the kernel. When these peaks coincide with those of $\mathcal{F}\{I_0(x, y)\}$, the l_1 norm reaches a maximum, which is identified as the location of maximum focus for the hologram. In other words, the sharp edges of the in-focus image represent a step function, in contrast to the smoothly varying intensity of an out-of-focus image (e.g. Bessel function). The spectrum of a step function consists of non-zero amplitudes at all frequencies, while a Bessel function has an associated bandwidth of frequencies. Due to the finite bandwidth, the Bessel function has a lower value of the l_1 norm compared to a step function at the same location, helping us differentiate between the two. Focus metric require only a single Fourier transform, whereas image-intensity-based methods require two. Therefore, focus metrics involve a significant reduction in the computational cost for longitudinal localization of objects, making it suitable for long-term tracking of fly behaviors.

Fly Arena

The fly arena consists of a custom made 70×35×50 mm^3 acrylic box (**Supplementary Fig.3**) with a specially designed feeding tray also made of acrylic. The tray has a food loading slot (30×25×1.5 mm^3) on one side and a feeding slot (30×1.5×2 mm^3) on the other accessible to the flies. SY 1:1 food prepared based on standard protocols[4] was loaded into slots and was replaced every 24 hours to maintain proper food quality. The imaging windows located in the path of the laser beam was designed to be replaceable to minimize the effect of scratches and other aberrations on the captured image.

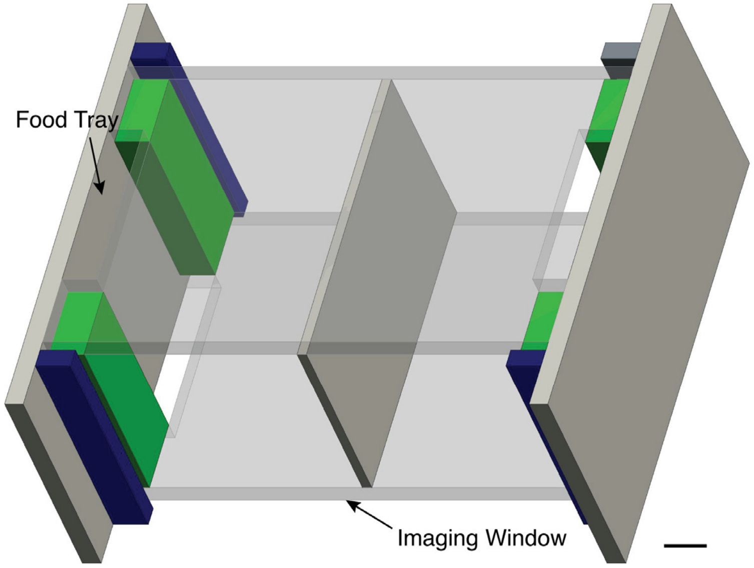

Supplementary Figure 3: 3D rendering of the fly arena used in the experiment, indicating the food trays and the replaceable imaging windows. Scale bar is 10 mm.

Estimation of Single Trajectories

The trajectories obtained after the planar tracking process contain several candidates consisting of multiple overlapped objects. To ensure long term statistics are calculated for single object, the algorithm automatically eliminates tracks with multiple objects overlapped. To identify the effectiveness of the algorithm, we compare the number of tracks remaining after elimination to the minimum possible number of single tracks. This count of possible single trajectories is estimated based on the number of objects detected at each time step after automatic thresholding. Using the number of flies in the arena we can specify the minimum number of the detected objects which can be single (**Supplementary Table 1**). For example, if the number of detections is 8, we know that we have one object that is merged with another, providing us 7 single objects at a minimum.

Supplementary Table 1: Estimated minimum number of single tracks based on detection

Number of Detections	Minimum Number of Single Flies that can be extracted	Comments
**9**	9	
**8**	7	single pair merged
**7**	5	2 pairs merged
**6**	3	3 pairs merged
**5**	1	4 pairs merged
**<=4**	0	

The total expected time of single tracks is calculated by multiplying the frequency of each detection count with the respective minimum number of single tracks and adding them up. Finally, we divide the sum by the maximum possible time corresponding to single tracks, assuming there are no overlaps i.e. total number of flies in arena × total duration of recording. The computation yields a value of 50%, which means that, for at least half the recorded time, the flies are overlapped/ interacting with each other.

Numerical Validation

The focus metric based on the maximization of the l_1 norm was validated, both through simulated synthetic holograms and positioning a needle at various distances using a linear translation stage. The synthetic test involves generation of holograms with square apertures located at specific *z* location and the calculation of the l_1 norm to identify the peak. The value of the norm increases close to the in focus location of the aperture and falls back down away from it.

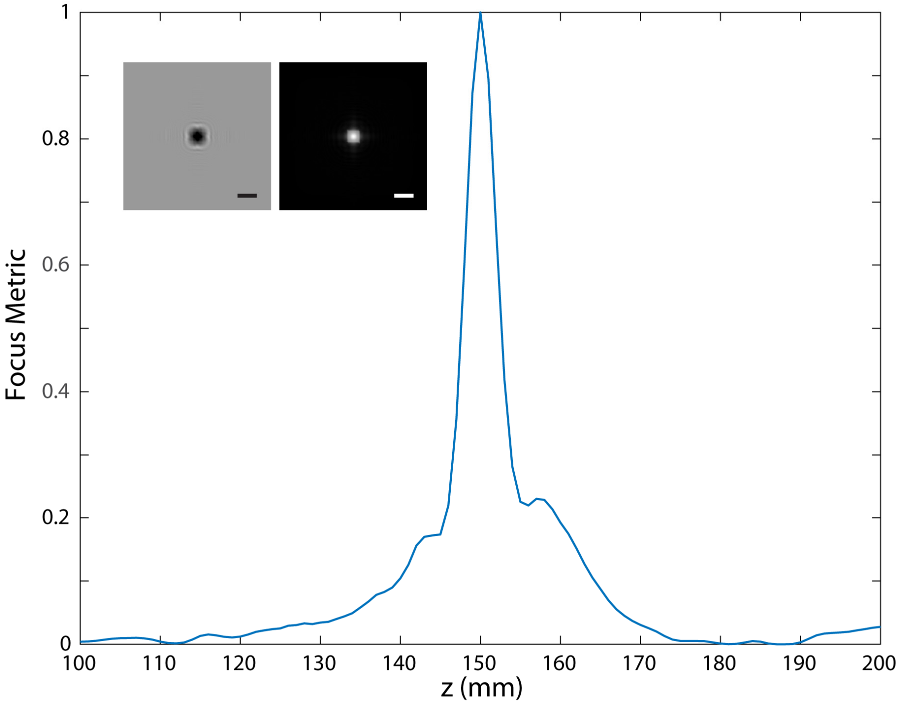

Supplementary Figure 4: A plot of focus metric with varying *z* positions. Insets are original and refocused hologram (inverted grayscale) of the synthetic aperture with 1mm scale bars.

The Fresnel kernel used for the reconstruction of the hologram places a limitation on the minimum *z* position that can be used which is based on the far field criterion[5] explained in a section below. So by placing an aperture at 150 mm from the imaging plane the

synthesized holograms satisfy this criterion and can be analyzed by the algorithm. The curve is then compared to the hologram reconstructed to each *z* position which clearly validates that the peak corresponds to the in focus location of the aperture (**Supplementary Fig. 4**). A detailed analysis of this phenomenon of minimum *z* limit is provided in the following section.

To validate the algorithm, we translate a hypodermic needle (460 μm diameter) on a linear stage (**Supplementary Fig. 5a**) and calibrate the displacement measured by holographic processing to the one set by the stage. The micrometer offers a precision of up to 10 μm which is much higher than the imaging resolution of our system. The experiment was conducted by first manually positioning the needle in the clamp (**Supplementary Fig. 5a**) and setting the micrometer to 0 mm. Then the distance is progressively increased in steps of 5 mm to 25 mm and holograms recorded at each position. We also capture a hologram without the needle present in the FOV for performing a background subtraction which improves the SNR of the captured fringes (**Supplementary Fig. 5b, c**). The images are cropped to a 256×256 ROI and processed using the focus metric algorithm, the curves for which are presented in **Supplementary Figure 5d**.

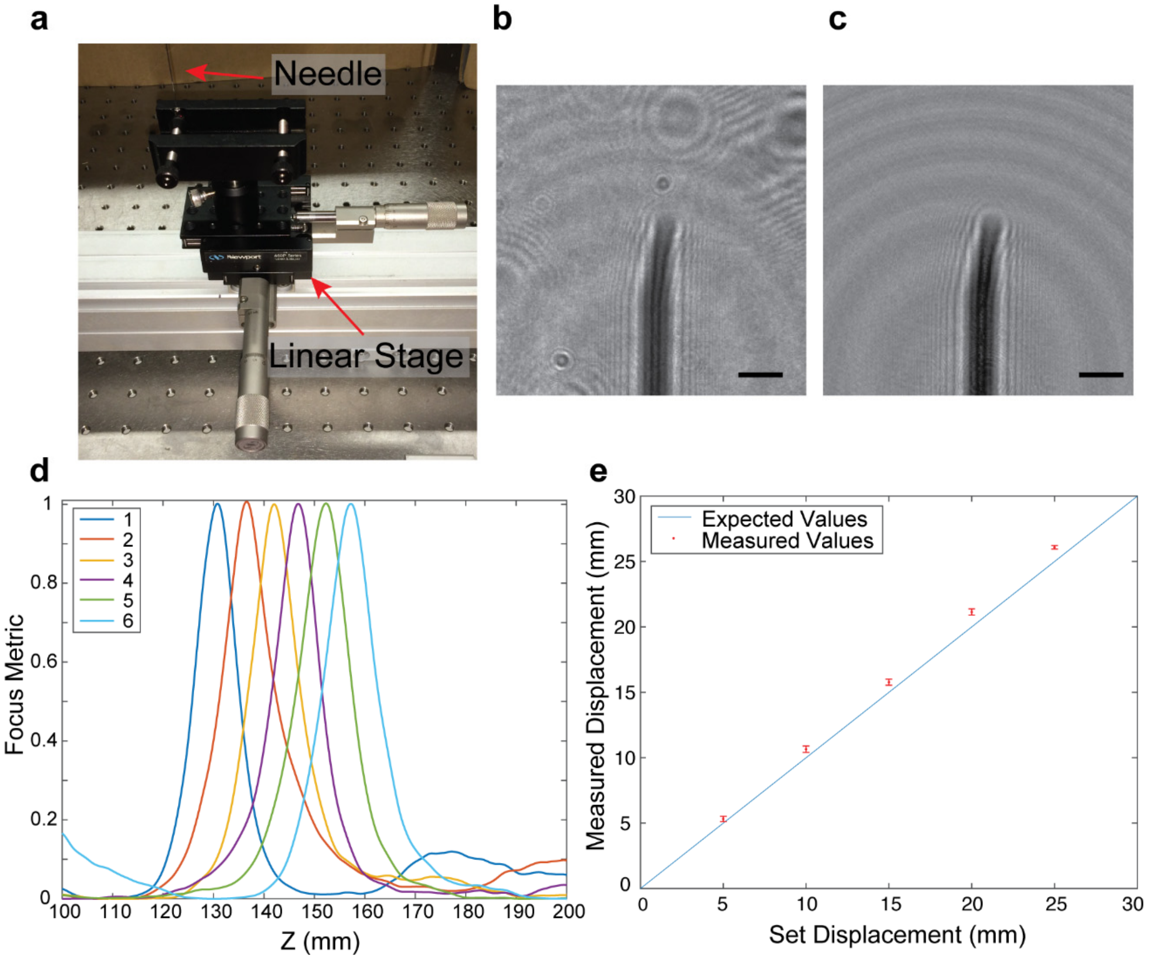

Supplementary Figure 5: (**a**) Image of the linear translation stage with a needle mounted to calibrate the focus metric method. (**b**) Original hologram of a needle with a scale bar of 1 mm. Note that some fringe patterns observed in the background of the hologram do not belong to the needle itself. (**c**) Enhanced hologram of needle with 1 mm scale bar. Almost all of background fringe patterns from **b** are removed which provides a higher SNR for the fringes of the needle. (**d**) Focus metric curves for the needle placed at 0 - 25 mm at 5 mm intervals marked as positions 1-6. (**e**) Expectation curve and the measured values of displacement with error bars showing the standard deviation from 7 trials.

The position of the needle at the zero of the stage is taken as a reference and the distance to all other positions from this is measured. The experiment is repeated seven times to measure the precision of the measurement. The mean difference of the measurement from the expected displacement is 0.796 mm with a standard deviation of 0.336 mm. A complete expectation curve with the mean value of the measurements and error bars of the standard deviation at each position are presented in **Supplementary Figure 5e**. To show that the peaks correspond to the accurate in-focus location of the needle, images of reconstructions at the peak location for one single experiment are showcased (**Supplementary Fig. 6**). It can be seen from this figure that due to inaccuracies in perfect alignment of the needle to be perpendicular to the camera, we see a few pixels of lateral motion. This motion corresponds to about ~300 μm for a 5 mm travel, which should also be included in the measurement uncertainty, though would have a negligible effect.

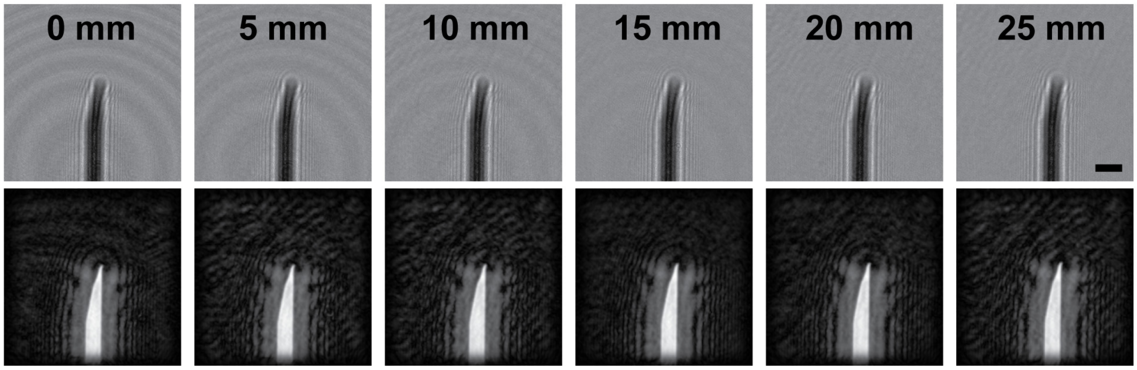

Supplementary Figure 6: Enhanced and refocused holograms (inverted grayscale to show better contrast) of all the positions of the stage with a scale bar of 1 mm.

In order to show that the focus metrics are independent of size, we repeated the experiment with a needle of 900 μm diameter and compared the errors and measurements

with the previous case (**Supplementary Fig. 7**). The focus metric curves for both images are of similar SNR, with small variations in the curve for the larger diameter (Case 2). The comparison of the measured displacements for both needles match within the measurement uncertainty obtained above (~300 µm). This shows that the accuracy and error in the technique doesn't vary with the size of the object and can be an effective calibration for our experiment involving flies with a body length of about 3 mm.

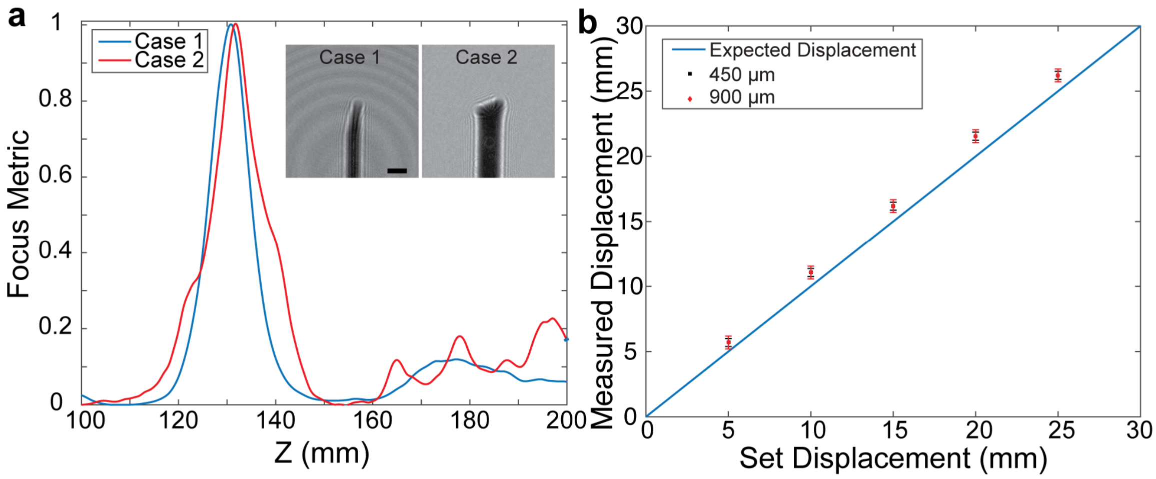

Supplementary Figure 7: (**a**) Plot of focus metric for the two separate needles of 460 µm (Case 1) and 900 µm (Case 2) diameters respectively at the reference position (0 mm). Insets are the enhanced holograms used as inputs with a scale bar of 1 mm (**b**) Expectation curve showing the variation of measured displacements with set displacement. Both samples (1 and 2) have a mean standard error of 300 µm and 500 µm respectively.

Automatic Thresholding

The histogram of the enhanced holograms (**Supplementary Fig. 8a**) shows a distribution with a peak at the background intensity and high frequencies at both ends corresponding

to the interior of the flies and saturations spots in the background. Most automatic thresholding algorithms expect a smoothly varying bi-modal or multi-modal distribution to be able to pick an accurate threshold to segment effectively (Otsu 1975). The current data set does not satisfy this requirement making its application difficult. An alternative and simple automatic threshold, equal to the mean of the 1st two peak values, was chosen to segment the hologram (**Supplementary Fig. 8b**). Such a threshold corresponds to pixels in the interior of the flies as well as some background pixels. Applying a mean filter before the segmentation and a morphological opening operation both with a disk of 10 pixels eliminates the segmented background pixels (**Supplementary Fig. 8c**). The opening operation also removes the legs and wings of the flies, which, if present, would increase the uncertainty of the *xy* position. Finally, a minimum size filter (greater than 500 Pixels) restricts segmented pixels that belong only to the flies. This automatic thresholding approach provides a robust method to identify the flies in the hologram the results for which are independent of the user.

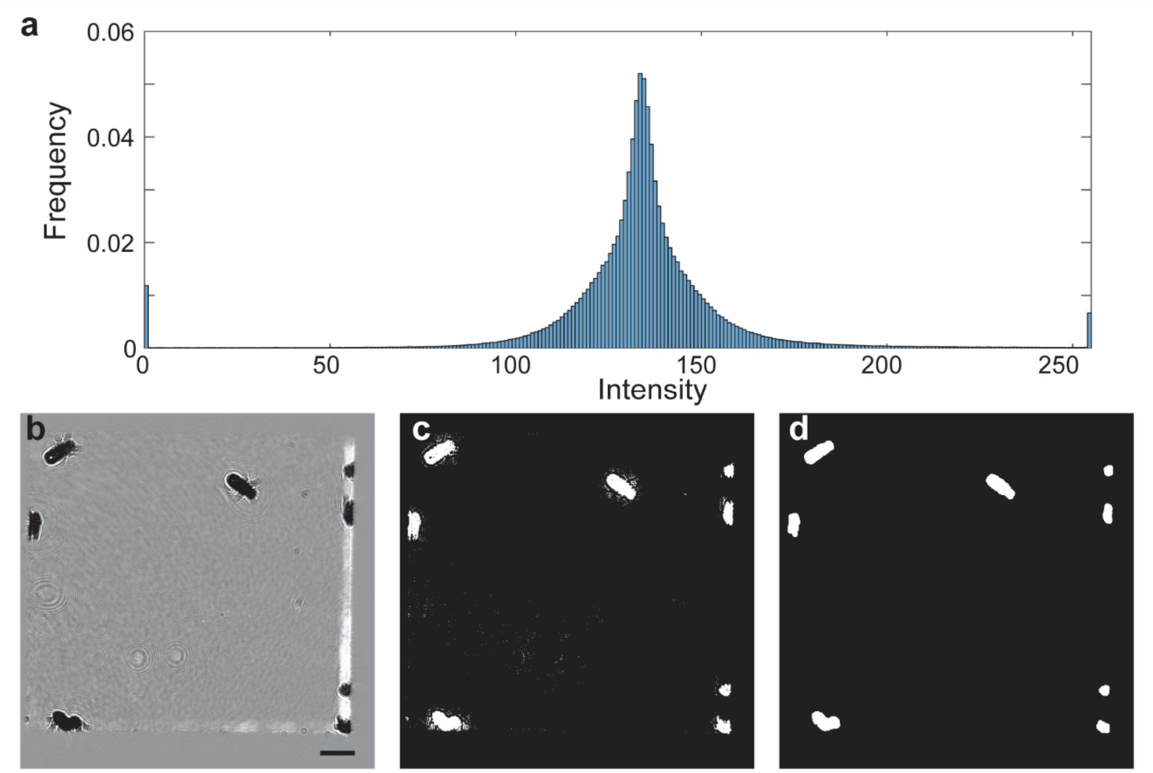

Supplementary Figure 8: (**a**) Histogram of enhanced holograms shown in **9b** with three distinct peaks at intensity values of 0, 134 and 255. (**b**) The enhanced hologram used to demonstrate the automatic threshold with a scale bar of 3 mm (**c**) Segmented image with the automatic threshold, showing a few noise pixels around the flies. (**d**) Final segmented image after the morphological opening operation.

Uncertainty Analysis

This section provides estimates of uncertainties in the measurements of position and velocity of *Drosophila* using the presented DIH approach.

Position Uncertainty

The uncertainty in position is provided for lateral and longitudinal directions respectively as follows: The lateral uncertainty involves the contribution from the automatic

thresholding, causing variations in the area segmented, and as a result of it the extracted centroids. The change in area over 500 time steps for stationary flies are plotted as a cumulative distribution and the 95% value is used to calculate the distance, modelling the area as a circle. We measured a value of 10 pixels which corresponds to 0.3 mm in each direction on the plane. The calibration with the needles provided the uncertainty in the measurement of z position for an object to be close to 0.8 mm (average difference between measurement and expected vales) and this value is independent of the object size. Thus the total uncertainty in position measurement would be ~ 0.9 mm, which is about 30% of the body length of the fly.

Speed Calibration

Similar to position, the error in estimation of the velocity consists of lateral and longitudinal contributions. The lateral motion of an object is obtained by tracking the diffraction pattern on the plane of the hologram. When the fly moves between two positions separated by distance Δx in time Δt, the velocity is calculated using first order finite difference scheme, i.e. $\Delta x/\Delta t$. The uncertainty in locating the fly due to the automatic threshold is found to be 10 pixels or ±0.3 mm. Provided that the relative uncertainty of timing is negligible compared with that of the position, the maximal velocity uncertainty is estimated as the first order finite difference scheme between the extremes of the uncertainty band around the two positions given by **Supplementary Equation 3**

$$v \pm \Delta v = \frac{\Delta x}{\Delta t} \pm \frac{2 * \sigma}{\Delta t}$$

Supplementary Equation 3: Estimate of velocity uncertainty in where σ_x is the measurement uncertainty in position.

Note that the uncertainty scales with the temporal resolution of the measurement, and a lower value of uncertainty can be obtained by reducing on temporal resolution.

The velocity measurement in the longitudinal direction is calibrated by translating a needle, 0.9 mm in diameter, at a constant speed along the optical axis, using a motorized linear stage (**Supplementary Fig. 9a**). A camera records holograms of this motion, at a resolution of 30 μm/ pixel at 100 fps, which are processed using the DIH algorithm to extract the *z* positions. The speed of the pump is measured through image based tracking, by recording the motion with a DSLR camera at 60 fps at a resolution of 125 μm/ pixel. By thresholding the captured video, we can segment a circle and identify its centroid, and use the position to extract the speed of motion (**Supplementary Fig. 9b**). We obtain a speed of 0.7424 ± 0.0036 mm/s where the uncertainty is from the thresholding operation calculated to be ±0.3 mm. The uncertainty in the measured position is converted into a velocity using the same equation (**Supplementary Equation 3**) as that in the lateral case. Similarly, the speed of the needle is also extracted from holographic processing by fitting a least squares line through the calculated positions (**Supplementary Fig. 9c**), providing us an estimate of 0.7331 ± 0.0188 mm/s. The source of uncertainty is from the linear fit used and the uncertainty in position measurement. The results indicate that the holographic measurement of speed matches the actual values within the measurement uncertainty.

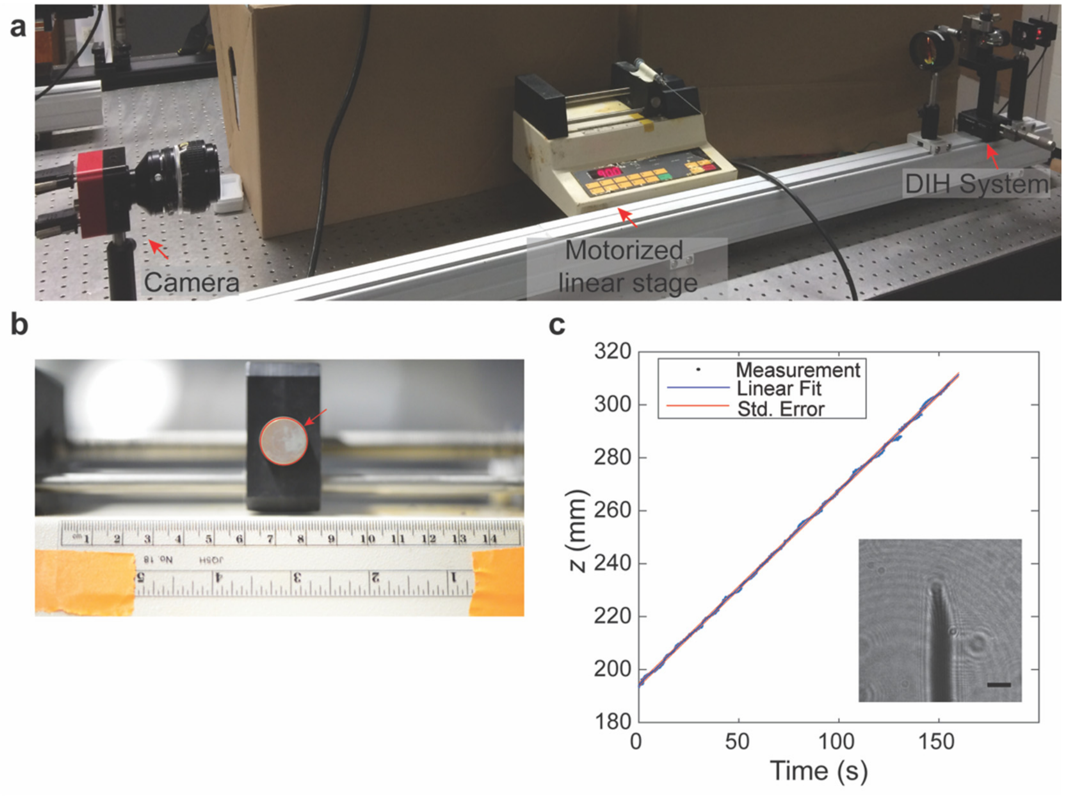

Supplementary Figure 9: (**a**) Experimental setup for calibrating the measurement of longitudinal velocity component using DIH. (**b**) A sample frame from the video used to calibrate the speed of motorized linear stage. The calibration is based on tracking the movement of the copper button (marked with a red circle and an arrow in the image). (**c**) Plot of z position vs time for the needle obtained through holographic processing. The inset is an original hologram recorded with a scale bar of 1 mm.

Multi-Pass Peak Selection and position correction

To eliminate small motions of flies, which are below the precision of our measurement (± 0.3 mm in xy and ± 0.8 mm in z) we perform a velocity based correction of position. For the lateral motion, we use a sparse sampled first order finite difference that ensures the displacement is greater than the uncertainty of the measurement. Using the lateral

velocities, we correct the position of the object at the next time step and this process is repeated over the entire track length in both *x* and *y* directions independently. To obtain the *z* position, we start with the values from the first pass of peak detection, which selects candidates based on the quality of the peak. During the first pass, a spline interpolation is applied on the focus metric functions and peaks with high quality are selectively sampled through the ratio of 1st two peak values ($P_1/P_2 > 10$). The interpolation eliminates the discretization of depth due to the step size used for reconstruction. The next step is to interpolate the missing *z* positions with low peak qualities and apply a similar sparse sampled velocity correction. A velocity corrected position is obtained in the longitudinal direction similar to the object's motion in the lateral plane. Due to the large scatter in extracted positions, we use the lateral velocity to place an upper limit on the longitudinal velocity to be of the same order or lower, assuming the statistics of motion to be similar in the *x-z* and *x-y* planes. In order to validate the accuracy of the Focus Metric peak selection algorithm, a specific motion sequence of a fly in the *z* direction was sampled manually from the data set and processed. We can see that there are several outliers in the *z* position (**Supplementary Fig. 10**), which arise from the presence of multiple peaks in the focus metric curves, which introduce error in the accurate positioning of the fly. The reasons for such low quality of peaks is explained in the following section, where we recreated similar profiles of the focus metric using the holograms of needles, used for calibration. We deal with such situations by applying a multi-pass approach to accurately select peaks and also avoid selection of spurious values in these scenarios. The results of the multi pass selection can be seen in **Supplementary Video 4** that contains the enhanced and refocused images together at each moment in time. Thus our algorithm can

effectively identify motion of objects even along the axis perpendicular to the imaging plane up to an accuracy of half the body size.

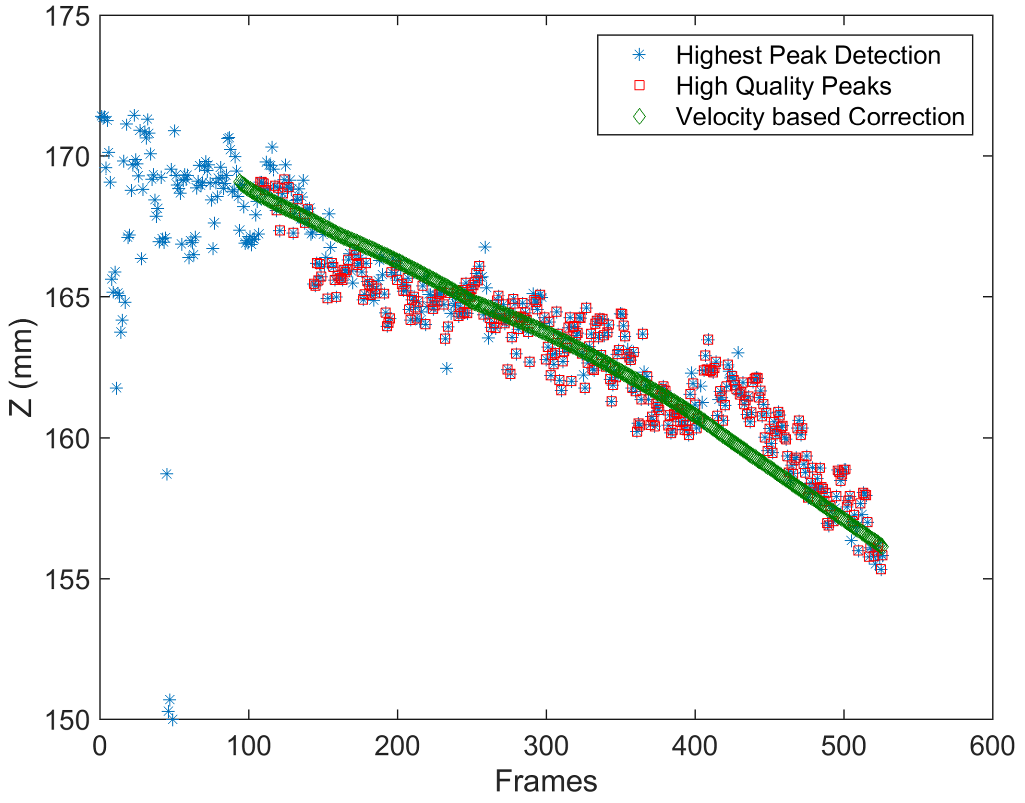

Supplementary Figure 10: Comparison plot of detected *z* position from primary peak selection and multi pass peak selection.

Poor Peak Quality

A systematic analysis is performed using holograms of the hypodermic needle, to identify the source of noise peaks in the Focus metric curves. Two specific cases are selected by changing the location of the region of interest (ROI) in the captured hologram, one with the tip of the needle and one without. The focus metric curves for both cases are created (**Supplementary Fig. 11)** in which we clearly observe a large noise peak near 100 mm,

for case 2 alone. Due to the absence of fringes in the horizontal direction, the needle from case 2 loses some longitudinal information that is contained in the horizontal fringes. This bias of fringe alignment leads to additional peaks in the focus metric curve of Case 2 compared with that of Case 1.

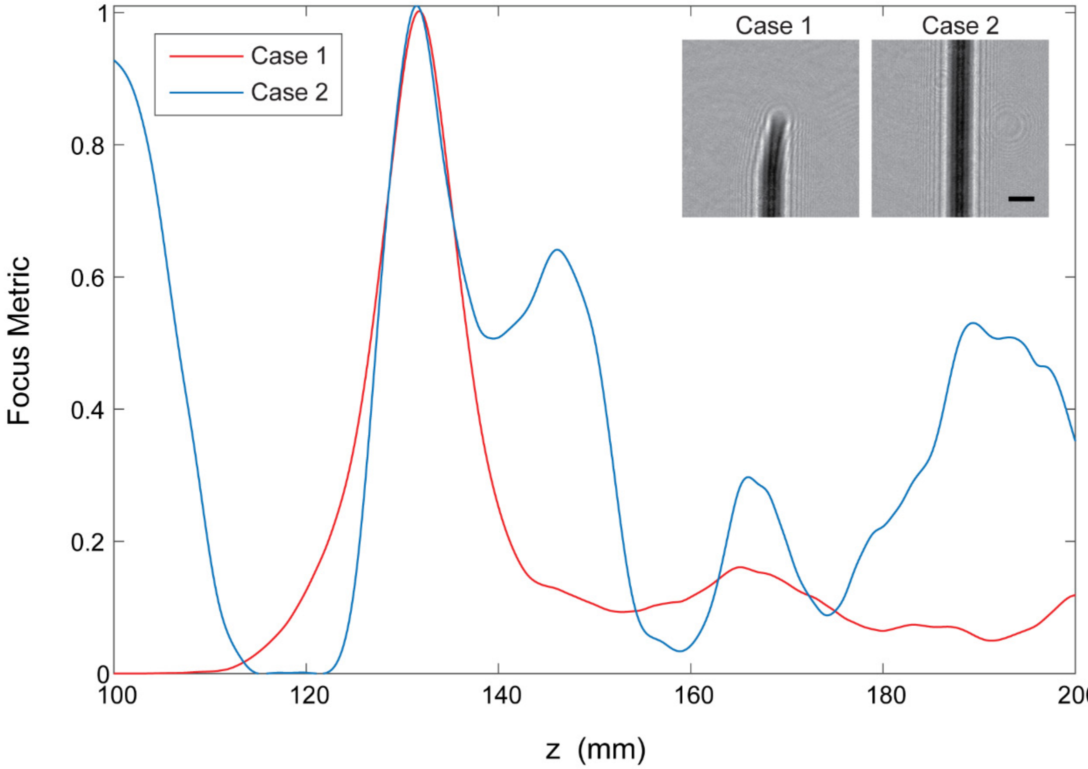

Supplementary Figure 11: Focus metric curves for 2 different ROI of the needle holograms which showcases the effect of bias in fringe alignment on the single-to-noise ratio. Inset are the holograms used for creating the focus metric plots with a 1mm scale bar.

The abovementioned scenario would arise when flies are located on any of the side walls, where the fringes of flies closer to the wall are suppressed by the wall-generated fringe patterns (i.e. horizontal or vertical). To demonstrate this point, we select three sample

holograms of flies from the recorded dataset with different relative position with respect to the wall. Specifically, the three cases, referred to as Case A, B and C, include a fly on the back wall and two on the bottom wall, for which the focus metric curves are calculated (**Supplementary Fig. 12a**).

Case A, where the fringe pattern associated with the fly are not contaminated by the wall generated fringes, show the most distinctive peak corresponding to the in-focus location of the fly (**Supplementary Fig. 12b**). Case B and C are samples with flies on the side and bottom walls, respectively (**Supplementary Fig. 12c** and **d**). Both samples have fringe patters that have been contaminated by the interference patterns of the walls resulting in focus metric curves with multiple prominent spurious peaks. The presence of multiple peaks makes selection of a maxima for the function difficult and thus cause the large scatter of z position observed in **Supplementary Fig. 10**. Currently, the issue of poor peak quality results in large variations in position (mostly unphysical displacements/accelerations), which is overcome through the post processing step. The post processing employs a speed-based filter to smooth the unphysical variation in speed due to the error of longitudinal positioning caused by the contamination of spurious peaks on the focus metric curve.

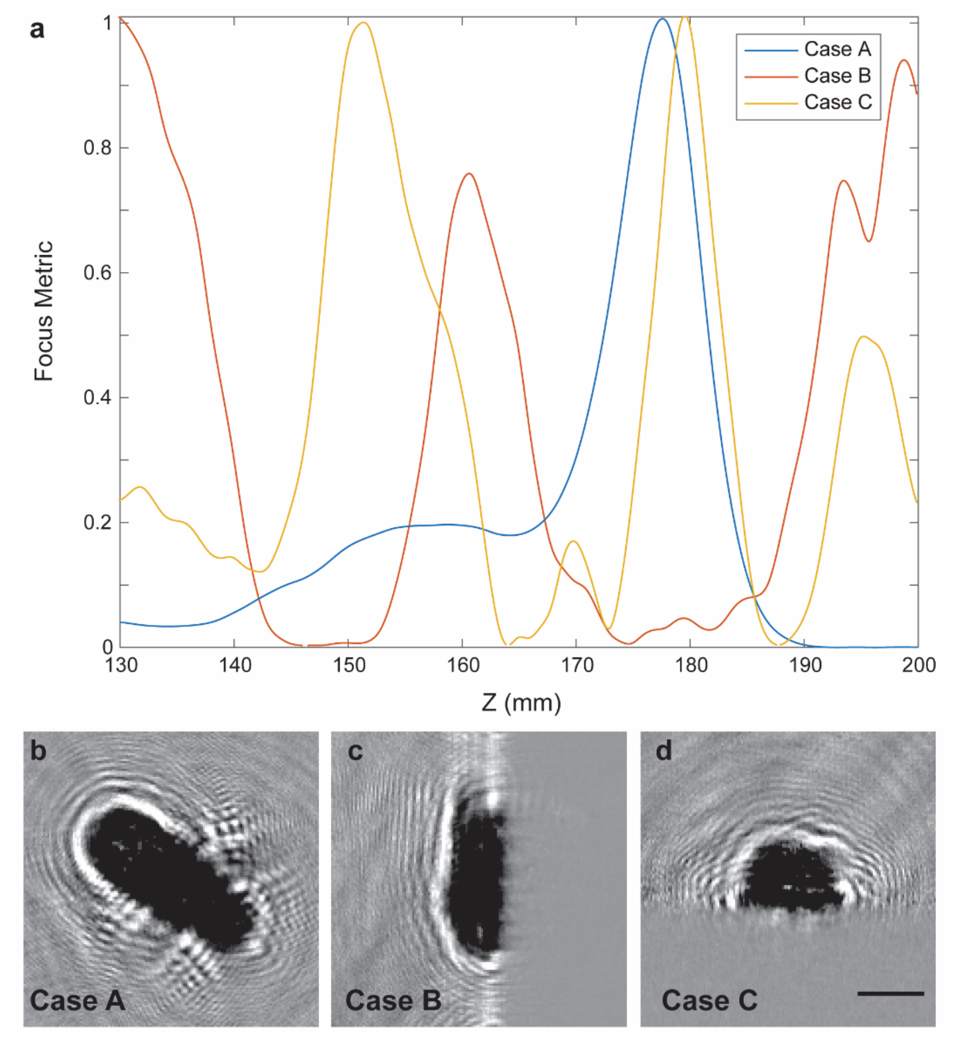

Supplementary Figure 12: (**a**) Plot of focus metric curves for 3 sample images to show the effect of asymmetric fringes. Sample holograms of flies on **(b)** the front wall, **(c)** the side wall and **(d)** the bottom wall with a scale bar of 1 mm.

Calibration of *z* position range for optimal performance

The location of the object affects the fringe patterns on the recorded hologram, and subsequently impacts the signal-noise-ratio of the focus metric curves. The calculation of the focus metrics is performed with the Fresnel diffraction kernel (**Supplementary Equation 4**), which is only valid when the optical imaging setup satisfies paraxial

approximation[5]. Specifically, as shown in **Supplementary Equation 5**, this approximation only holds for the case that the axial distance of an object to the recorded hologram plane is significantly larger than its lateral distance to the optical axis. Consequently, as the axial distance between the object and the recording plane (z) approaches zero, the functional form of the kernel represents a delta function at the origin (i.e. $z = 0$) and overwhelms the calculated l_1 norm close to this location.

$$H(f_x, f_y, z) = \exp\{-i\pi\lambda z(f_x^2 + f_y^2)\}$$

Supplementary Equation 4: Fresnel Diffraction kernel with the quadratic phase given by the square terms

$$z^3 \gg \frac{\pi}{4\lambda}(x^2 + y^2)^2{}_{max}$$

Supplementary Equation 5: Condition for the validity of the paraxial approximation, where λ is the wavelength of light source and (x, y) is the position of the object on the hologram relative to the center.

Based on the current imaging system (632 nm illumination) and size of the fly on the hologram (1.5 mm on the lateral plane), the paraxial limit is identified as $z >> 28.3$ mm. In order to illustrate the effect of the delta function and identify a suitable position for the fly arena, we recorded holograms of a needle (900 µm diameter) at specific distances (50 mm to 250 mm in 50 mm increments) from the in-focus plane. The holograms are cropped to 100×100 pixels and processed through the focus metric method using two different z ranges as inputs. For the first case the computation is carried out over 0 to 275 mm. The peaks corresponding to the location of the needle are overlapped by the strong signature of the delta function at the origin which greatly suppresses individual peaks

(**Supplementary Fig 13a**). However, when we start the *z* scan at 90 mm (three times *z* limit) and end at 275 mm, the large peak at the origin is completely suppressed and the individual peaks become appreciable (**Supplementary Fig. 13b**). In order to avoid the effects of the delta function at the origin, during the fly experiments, we set the location of the arena to be 140 mm, which is 1.5 times of the minimal recorded distance for implementing the focus metric method in the present study.

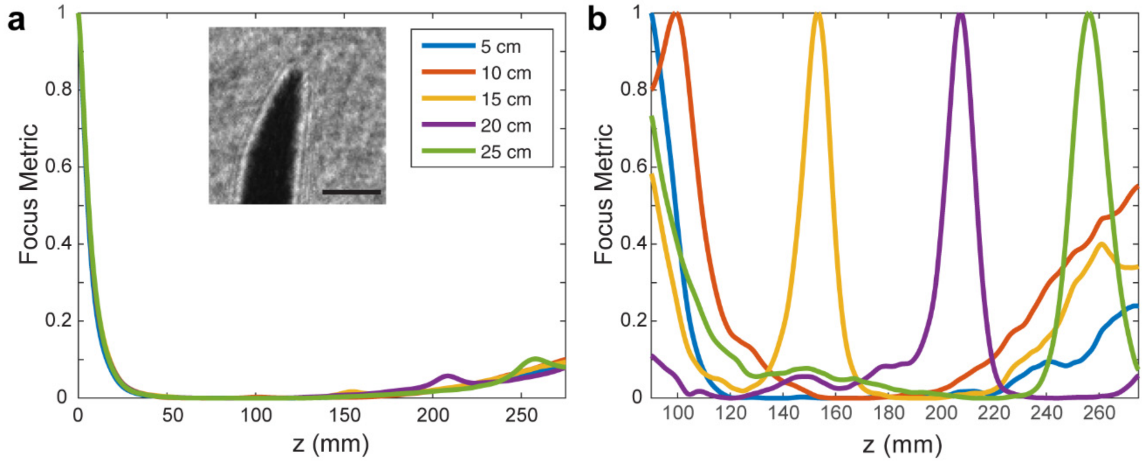

Supplementary Figure 13: Focus Metric plots of a needle located at different distances from the hologram plane. (**a**) When reconstructed from a minimum distance of z = 0 mm showing the large peak near the origin and smaller peaks at 150, 200 and 250 mm, respectively. The inset is a sample of the recorded hologram with a 1 mm scale bar. (**b**) The same hologram processed from a minimum distance of 90 mm showing distinct peaks for the four locations (100, 150, 200 and 250 mm) within the range of processing. The same legend is used for both figures.

Ethograms

The ethograms of motion are created for the three selected trajectories with three specific levels of motion defined as resting, walking and flying. These are identified by a threshold of speeds computed for each trajectory and can be seen in the **Supplementary Table 2** below.

Supplementary Table 2: Speed Threshold for Ethogram Generation

Motion	Threshold
**Resting**	0 < speed < 3 mm/s
**Walking**	3 < speed < 17 mm/s
**Flying**	17 < speed < ∞ mm/s

The calculated speeds are filtered with a median filter of size 5 to eliminate large fluctuations in speed that arise at locations of large acceleration (quick turns). Along with the speed, position of objects in proximity to the side walls can also be used to define walking motions.

Identification of complex motions

The complex behaviors we identify in this study are landing responses, consisting of high speed motions. Using the speed of the fly calculated at every instant of time, an identification function is created to separate trajectories with complex behaviors from the complete list, before the track elimination step (**Supplementary Equation 6**). The

identification of tracks before elimination ensures that complex motions with partial occlusions in the motion are not missed. A threshold of mean plus twice the standard deviation on the function helps identify 25 tracks with complex motions from the list of 5700, corresponding to 0.44% of the total. The plot in **Supplementary Figure 14** represents the values of this function for all selected tracks whose color indicates whether the selection was accurate or a false positive.

$$F = \max\{v\} * (\max\{u\} + \min\{u\})$$

Supplementary Equation 6: Vertical motion identification function

After identifying specific track IDs, the velocity and acceleration of those specific tracks over time are used to narrow down the time of the motion. We create in-focus movies of these specific sequences (**Supplementary Video 1 & 2**) by the holographic processing algorithm that additionally saves refocused images as a video at the resolution images were recorded at. With an increased data set additional machine learning approaches can be applied to make the identification process more robust.

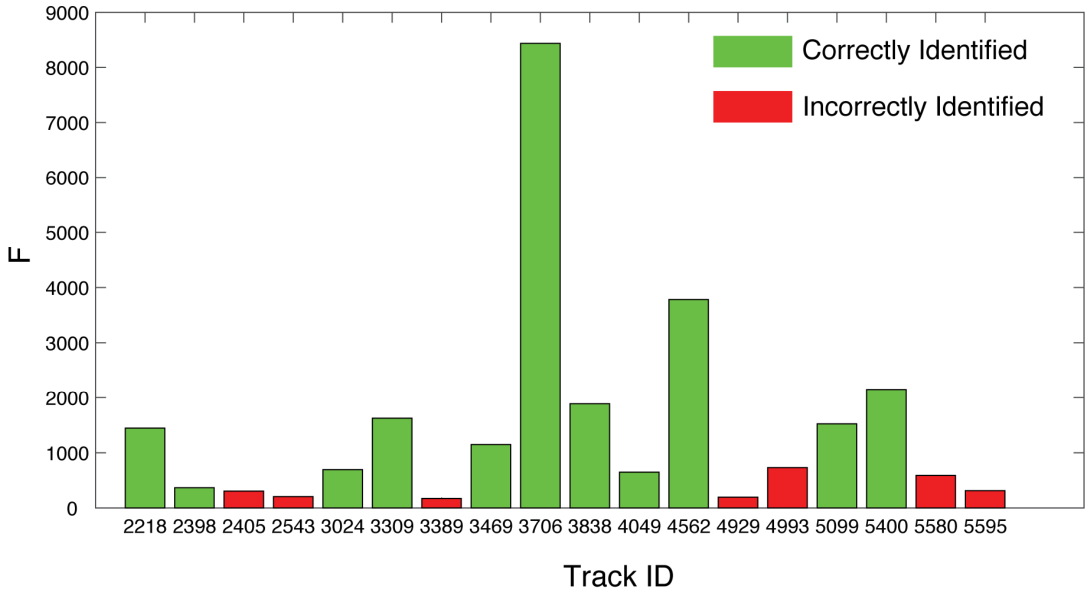

Supplementary Figure 14: Plot of F for the tracks selected by the algorithm. The green bars correspond to tracks that correctly identified the motion in the vertical direction and the red ones are false positives.

Pseudo Algorithm

Image Enhancement:

For all images in sequence

Sum all images to create background (BG)

For all images in sequence

Subtract BG from image and rescale the result to occupy 0-255

end for

Segmentation:

For all images in sequence

Calculate histogram

Calculate automatic threshold (mean of 1st 2 peaks)

Mean filter of size 10 pixels

Threshold image

Fill holes

Morphological opening with 10-pixel disc

Filter objects based on area and number of objects in arena

Save the position and area of objects

end for

Tracking: Tracking was performed using code published by Wilkinson et al. 2014 used for tracking mosquitoes.

After this step we split into 2 parts: long term statistics, complex behaviors:

Long term statistics:

Track Elimination

For each time step

Calculate the number of tracks present

If total number equals number of objects

Identify all tracks as single/valid

Else if number of tracks decreases from previous step

Mark Merging event

Mark tracks present at previous time & missing now

Use distance between tracks and area change to find which trackids combine to form which trackid

Else if number of tracks increases from previous time and number equals total number of objects

Mark Emerging event

All tracks are marked as single

Use distance between tracks and area change to find which trackid split to form which trackids

Else if number of tracks increases from previous time

Mark Emerging event

Use distance between tracks and area change to find which trackid split to form which trackids

End if

end for

For each merging event

Mark the source trackids as ending/not single/not valid after the current time step

Identify destination as merged tracks

Keep track of objects merged into it

```
        Increase count of objects in track by 1
end for
For each emerging event
        If distance between the new trackid from the source > body length (150 Pixels)
                Mark object as single/valid
                Reduce count of objects in partner track by 1
        Else if less
                Mark object as merged
                Reduce the count of objects in partner track by 1
        End if
end for
```

Reconstruction (Based on Li et al 2009**)**

```
For all time steps
        If track is valid
                Crop ROI around centroid of size 150 pixels
                Multiply the image by a tukey window to eliminate aliasing during
                calculation of FFT
                Calculate FFT of hologram with zero pad up to 256 pixels
                Multiply FFT by a Gaussian window (alpha = 3) as a low pass filter
                For all z values in 1 mm increments
                        Calculate Fresnel kernel
                        Calculate first focus function as l1 norm of FFT of Hologram × real
                        {kernel}
                        Calculate second focus function as negative of l1 norm of FFT of
                        Hologram × imaginary {kernel}
                end for
                Detrend both functions to remove the linear trend (detrend function)
                Smooth the function over 3 samples with a mean filter
                Multiply the functions to increase the contrast of Focus Metric
                Save value of function
        End if
end for
For all focus metric curves
```

Interpolate by spline with 0.01 mm spacing

Identify peaks and save location + peak value

End for

For all focus metric curves

If ratio of 1st to 2nd peak > 10

Save *z* position (1st pass)

End if

End for

Post Processing

For all focus metric curves

If length of track > minimum limit

For all time steps in *x*/*y*

Calculate velocity by sparse sampling above uncertainty limit (*x* & *y*)

Use velocity to predict the position at next time step

End for

For all time steps in *z*

Median filter 1st pass *z* values (width 5)

Linearly Interpolate missing values

Calculate velocity based on sparse sampling

Correct position based on velocity

Add count to 3D histogram bins to calculate spatial distribution

Save 3D position of tracks

End for

End for

Calculate distance of nearest object

Export to histogram

Create ethograms based on velocity thresholds

Complex Motions:

For all tracks

Calculate the u and v velocity

Compute the function F to identify motions of interest

Select tracks based on threshold of F

Save time stamp of beginning of motion + length of motion

End for

For all candidates of motion

Perform tracking using same code but over length of motion

Identify flying or falling motions from tracks

Perform reconstruction using focus metric algorithm

Save reconstructed images as a video

End for

Legends for Supplementary Videos

Supplementary Video 1 Recorded Hologram of multiple drosophila and in focus reconstruction of the falling drosophila with embedded timestamps.

Supplementary Video 2 Recorded Hologram of multiple drosophila and in focus reconstruction of a landing drosophila with embedded timestamps.

Supplementary Video 3 2D Trajectories of single drosophila detected after the post processing step rendered with colors representing their motion speeds. The black square indicates the ROI and the circle the location of the fly in the hologram selected.

Supplementary Video 4 Recorded hologram and in focus reconstruction of a single drosophila with dominant motion in the longitudinal direction. This sample was used for validation of the post processing algorithm in capturing motion along the longitudinal direction.

References

1. Kreis T. *Handbook of Holographic Interferometry: Optical and Digital Methods.* John Wiley & Sons; 2006.

2. Hansard M, Lee S, Choi O, et al. *Time of Flight Cameras : Principles , Methods , and Applications*.; 2012.

3. Li W, Loomis NC, Hu Q, Davis CS. Focus detection from digital in-line holograms based on spectral l1 norms. *J Opt Soc Am A Opt Image Sci Vis*. 2007;24(10):3054-3062. doi:10.1364/JOSAA.24.003054.

4. Sun Y, Yolitz J, Wang C, Spangler E, Zhan M, Zou S. Aging Studies in Drosophila melanogaster Yaning. In: *Biological Aging*. Vol 1048. ; 2013:323-352. doi:10.1007/978-1-62703-556-9.

5. Goodman JW. Introduction to Fourier Optics. *Quantum Semiclassical Opt J Eur Opt Soc Part B*. 1996;8(5):491. doi:10.1088/1355-5111/8/5/014.
